# Microbiome: A New Perspective on Immunotherapy for Metastatic Tumors

**DOI:** 10.1002/mco2.70185

**Published:** 2025-05-01

**Authors:** Hongquan Liu, Tianqi Wang, Jitao Wu

**Affiliations:** ^1^ Department of Urology The Affiliated Yantai Yuhuangding Hospital of Qingdao University Yantai Shandong China

1

A recent study in *Cell* by researchers from the Netherlands Cancer Institute used multi‐omics methods to explore the microbiome of metastatic tumors [1]. Analyzing over 4000 biopsy samples, they found correlations between microbial composition and tumor immunity and responses to immunotherapy. This comprehensive resource highlights the connection between tumor immunity and microbial dynamics, contributing to the development of immunotherapy strategies.

With the advancement of sequencing technology, there is a growing recognition of the vast array of symbiotic microorganisms that reside within various organisms. These symbiotic microbes exhibit intricate dynamic interactions with the host's immune system and are involved in various physiological processes. Recent research on symbiotic microbes has shifted our focus to the species and functions of tumor‐associated microbes [2]. The intratumoral microbiome has been demonstrated to not only directly act on tumor cells, promoting or inhibiting their growth, but also indirectly regulate the immune escape mechanisms of tumors by influencing immune cells within the tumor microenvironment. Studies have revealed that gut microbiota can modulate the response to immune checkpoint blockade (ICB) therapy and traditional chemotherapy, as well as reshape the tumor microenvironment through microbes residing in primary tumors [3]. By offering a detailed fingerprint of an individual's immune status and responsiveness, the microbiome presents a transformative opportunity for personalized medicine. Based on unique microbial signatures, clinicians can customize immunotherapy to individual patients, enhancing outcomes, minimizing adverse effects, and improving quality of life and survival rates for metastatic tumor patients. However, numerous scientific questions remain to be addressed. For instance, the characteristics of the microbiome in metastatic tumors are still undetermined. Do the microbiomes of primary and metastatic tumors exhibit differences? Is the colonization of microbial species determined by tumor type or organ type? Additionally, the evolution of microbial populations during therapeutic interventions is also uncertain.

In Battaglia's work, researchers conducted a thorough analysis of 4160 metastatic tumor biopsy samples from 26 different tissues, using Kraken2, PathSeq, and an assembly‐based approach along with corresponding genomics and transcriptomics data to precisely identify and quantify microbial communities within the metastatic tumors. This study revealed that bacterial DNA could be detected in metastatic tumors, and its diversity displayed a close correlation with tumor immune characteristics at the cellular and molecular levels. Among the findings, metastatic colorectal tumors exhibited the greatest species diversity, while head and neck metastatic tumors harbored a greater abundance of dominant bacteria.

Notably, what factors shape the microbiome composition of metastatic tumors? Firstly, the authors discovered that the anatomical location of biopsy lesions has a dominant impact on the composition of microbial communities. Secondly, their theory proposes that hypoxic tumors may provide a favorable environment for a higher abundance of anaerobic bacteria. To validate this hypothesis, the researchers utilized a set of paired RNA‐seq data from metastatic tumors and an external human papillomavirus‐negative head and neck tumor cohort. Lastly, they found that genomic alterations might drive the microbial community composition with preferences for different cancer phenotypes, with microsatellite instability (MSI) status being associated with distinct microbial clusters.

After clarifying the above issues, the authors delved into the impact of microbial communities on the host tumor microenvironment. The study revealed extensive correlations between bacterial components and tumor gene expression. Simultaneously, the authors noted a positive correlation between microbial diversity and the signaling expression of immune exclusion and cancer‐associated fibroblast infiltration. Tumors exhibiting greater microbial diversity were found to harbor an abundant presence of congenital immune cells, including neutrophils, natural killer cells, macrophages, and Tregs. These findings indicate a close relationship between the immune system and the microbiome and their significant role in shaping the tumor microenvironment.

Interestingly, the authors also observed that the composition of microbial communities in metastatic tumors evolves as the tumor progresses. Moreover, immunotherapy affects the microbial composition in tumors, with a notable reduction of bacterial diversity among individuals undergoing ICB treatment. In a subset of patients, those who responded well to immunotherapy exhibited significantly lower bacterial richness compared to nonresponders. The authors argue that immunotherapy exerts a targeted impact on the microbiome of metastatic tumors, reshaping the community structure, which is more significant in responsive individuals. Analysis of lung cancer patients with a low response to ICB revealed higher abundances of Fusobacterium in their tumors, which strongly correlates with diminished efficacy of ICB in metastatic non‐small cell lung cancer. These findings in the tumor microbiome underscore the intricate interplay between microbial communities and cancer immunotherapy. The gut microbiome exhibits the greatest diversity of microbial species. Previous research has reported that the gut microbiome can regulate responses to ICB and traditional chemotherapy and is associated with molecular mimicry of tumor neoantigens. Decreases in diversity observed in conditions such as antibiotic use, dietary changes, or disease states disrupt the delicate balance of the gut microbiome, leading to immune dysfunction and altered treatment responses [3].

While this study provides a detailed and extensive pan‐cancer resource on the microbiome of metastatic cancers, previous research has shown significant differences in the microbiome between healthy controls and primary tumors [4]. Since this study only comprised metastatic lesions, it does not permit a direct evaluation of differences from healthy communities. Additionally, it insufficiently considers the impact of confounding factors (such as patient heterogeneity and environmental influences) on the conclusions. Despite the numerous advancements in tumor microbiota research through omics methodologies, this field is still in its infancy. Many research techniques are still maturing. Researchers like Galeano Niño have uncovered the spatial, cellular, and molecular interactions between the host and intratumoral microbiota based on a novel single‐cell RNA sequencing methodology called INVADEseq (invasion–adhesion‐directed expression sequencing) [5]. Consequently, the integration and utilization of multi‐omics technologies in microbiota research will further elucidate the interactions of microbiota in tumor development across temporal and spatial scales. Furthermore, this study is a descriptive report of phenomena based on data, and further experiments are still required to thoroughly validate the predicted functions and explore the underlying biological pathways. Establishing a clear pathway from research findings to clinical application is also a crucial challenge that must be addressed in the future.

Overall, by integrating metagenomics, genomics, and transcriptomics, this article analyzed and described sequencing data from 4160 metastatic tumor samples. The study revealed the distribution and diversity characteristics of DNA in species‐level resident microbiomes of metastatic tumors on a pan‐cancer scale, enriching researchers' insights into the microbial features of metastatic tumors. These data will serve as a valuable resource for future in‐depth studies exploring the potential therapeutic targets in metastatic cancer. Simultaneously, this research dissected the evolution of microbial composition during tumor immunotherapy, revealing the impact of Fusobacterium abundance on immunotherapy outcomes in non‐small cell lung cancer, emphasizing the correlation between tumor immunology, microbial community dynamics, and immunotherapeutic effect (Figure 1). Battaglia's work provides new theoretical and experimental evidence for novel alternative clinical strategies for metastatic tumors. The integration of tumor microbiomics with various omics and technologies, including autoantibody detection, circulating tumor DNA sequencing, and innovative imaging modalities, holds the promise of unleashing its transformative potential in cancer therapy.

**FIGURE 1:**
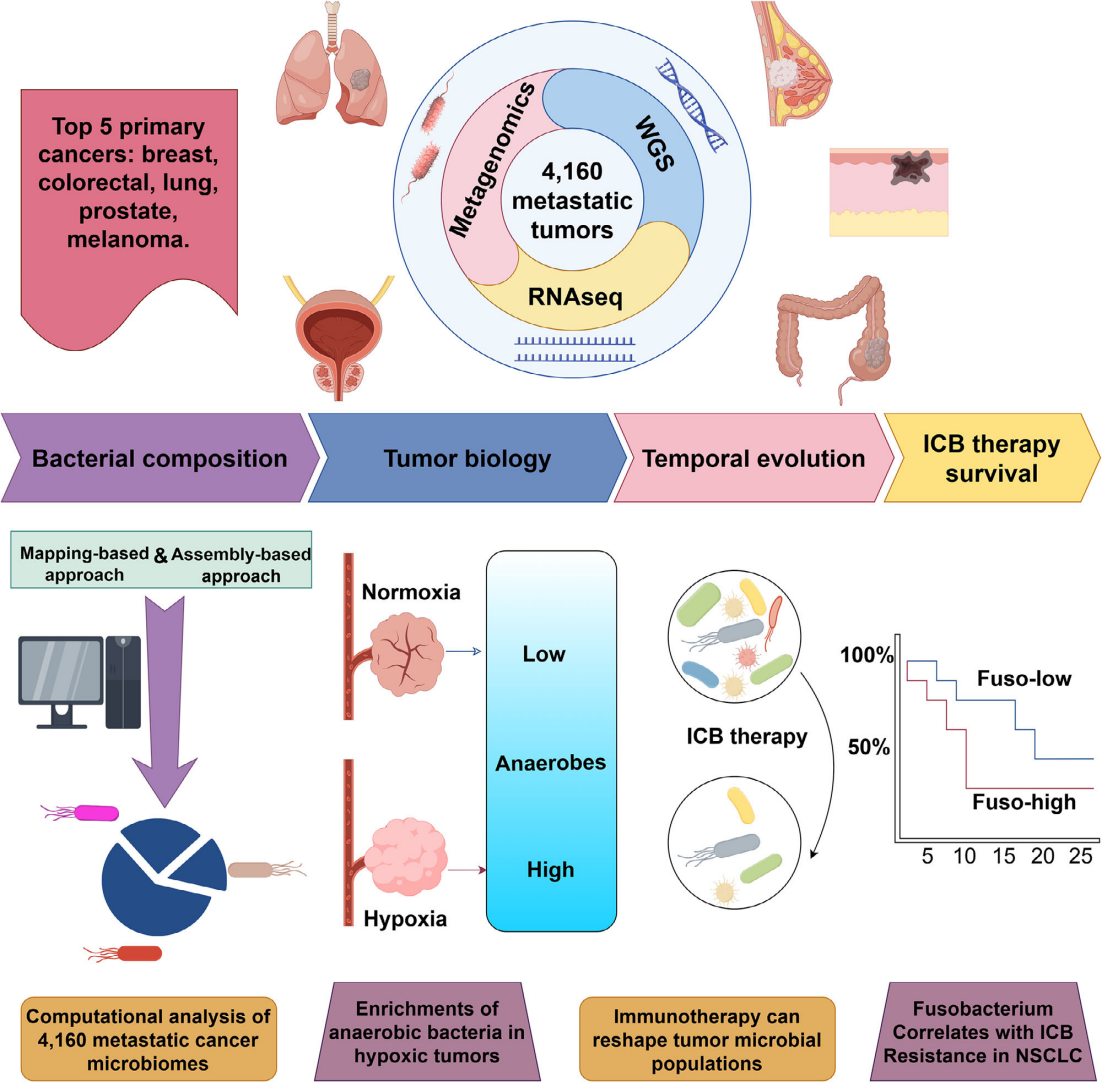
The current study integrated mapping and assembly‐based metagenomics, genomics, transcriptomics, and clinical data from 4160 metastatic tumor biopsies to investigate the characteristics, evolution, and clinical relevance of the microbiota in metastatic tumor samples. Through detailed annotation and characterization of intratumoral microbiota, the authors revealed the enrichment of anaerobic bacteria in hypoxic tumors and elucidated the evolution of microbial composition during immunotherapy, observing a significant decrease in bacterial richness in patients receiving immune checkpoint blockade (ICB) treatment. Furthermore, the authors explored the microbiota associated with poor response to ICB therapy and found a strong correlation between the presence of Fusobacterium and a poor response to ICB in metastatic non‐small cell lung cancer. ICB, immune checkpoint blockade; WGS, whole‐genome sequencing.

## Author Contributions

H.L. wrote the manuscript with critical reading and feedback from T.W. J.W. guided the literature research and edited the manuscript. All authors have read and approved the final manuscript.

## Ethics Statement

The authors have nothing to report.

## Conflicts of Interest

The authors declare no conflicts of interest.

## Data Availability

The authors have nothing to report.
